# The Relationship between Bicultural Acceptance Attitude and Self-Esteem among Multicultural Adolescents: The Mediating Effects of Parental Support

**DOI:** 10.3390/ijerph21091175

**Published:** 2024-09-04

**Authors:** Youn-Joo Um

**Affiliations:** Department of Nursing, Dongyang University, Yeongju 36040, Republic of Korea; digimong8333@gmail.com

**Keywords:** cultural diversity, self-esteem, adolescents, parents, social status

## Abstract

As the rate of international marriage increases, the adaptation of multicultural families and their children—multicultural adolescents—remains an issue requiring continuous monitoring, support, and improvement. This study investigates the mediating effects of parental support on the relationship between bicultural acceptance attitude and self-esteem among multicultural adolescents. Data are derived from 1197 high school sophomores, all of whom were multicultural adolescents, surveyed by the National Youth Policy Institute in 2018. Descriptive statistics, correlation, and hierarchical regression were used during data analysis. Bicultural acceptance attitude, parental support, and self-esteem all showed mutual positive correlations. Parental support had a partial mediating effect on the relationship between bicultural acceptance attitude and self-esteem. Multicultural adolescents exposed to both parent’s cultures are more likely to develop positive self-esteem and a healthy personality if they receive the support of their parents. The self-esteem of multicultural adolescents can be improved by implementing measures to nurture a bicultural acceptance attitude and parental support.

## 1. Introduction

As migration between ethnic populations increases, conflicts arising from immigrant culture and population movement are emerging as a significant global issue. Therefore, every country must develop modern multiculturalism policies [1]. In Korea, international marriages initially gained popularity during the 1990s, mainly among single men in rural areas who were past marriageable age. After the establishment of diplomatic relations with China, many ethnic Korean women came to Korea, and there are many cases of Filipino women marrying Korean men in search of better living conditions. Following the feminization of migration in Korean society, most multicultural families in Korea involve marriages between Korean men and foreign women. More than 70% of married immigrant women emigrated from Southeast or Northeast Asia. The fact that most of these women emigrated to provide economic support to their families in their home countries is a characteristic of Korean multicultural society that is different from Western societies. While the number of domestic marriages and related births in South Korea has decreased in recent years, the proportion of multicultural marriages and the number of youths born into multicultural families have increased [2].

Given this rapid increase in multicultural families, the healthy development of multicultural adolescents is emerging as a considerable social concern [3]. In addition, adolescents from multicultural families may face additional problems of cultural adaptation as a minority race or ethnicity during the delayed stage between childhood and adulthood. Adolescents experience considerable stress because of the developmental tasks they must accomplish during this stage in life [4]. The formation of self-awareness—including self-esteem (the degree to which a person understands themself to be capable, meaningful, and valuable)—is a primary developmental task of adolescence [5]. Adolescents become confused if they fail to develop confidence in their own worth [6]. In addition, greater self-esteem will positively affect the development of a healthy personality, self-belief in one’s abilities, and actual achievements [7]. For multicultural adolescents, the background variable of culture can act as a significant stress factor. The multicultural background as a minority of a minority race can have a negative impact on the individual’s self-esteem, and as a marginal being rather than a major being, they can experience psychosocial stress due to the sense of alienation between the two cultures.

Multicultural adolescents must adapt to the different cultures of two parents [8], and the resulting cultural conflict and confusion in their cultural identity could lead to increased difficulty in forming self-esteem [9,10]. Bicultural acceptance refers to an attitude that embraces two cultures without prioritizing either one [11]. For a multicultural adolescent in Korea, this means accepting both Korean culture and the culture of the foreign parent; ultimately, bicultural acceptance positively affects school adaptation, mental health, and happiness [12]. Studies indicate that greater bicultural acceptance leads to better friendships, better adaptation to school life, and less sensitivity to discrimination [13].

Multicultural adolescents who positively accept biculturalism will acquire the language and culture of both parents and develop an ability to respond flexibly to culture [14]. In contrast, those adolescents who do not accept biculturalism have been found to experience cultural stress and identity confusion [15]. Prejudice against race and culture among parents affected prejudicial attitudes in their children [16]. Studies have reported that compassionate and positive parenting attitudes in bicultural families increase biculturalism and positive perceptions of biculturalism among children [17].

Previous studies have demonstrated that positive parenting attitudes positively affect adolescent acceptance of multiculturalism and that a multicultural adolescent’s self-esteem positively affects their multicultural acceptance [18]. However, there is a dearth of experimental considerations regarding how parental support and bicultural acceptance affect self-esteem formation, a primary developmental task for multicultural adolescents [19,20,21]. Thus, this study aims to verify whether parental support mediates the relationship between bicultural acceptance attitude and self-esteem among multicultural adolescents. Specifically, this study tests whether parental support will positively mediate a multicultural adolescent’s bicultural acceptance attitude and self-esteem.

Since multicultural families in Korea are composed of young female immigrants from Asia, such as those from China, Japan, and the Philippines, and relatively older Korean males, this study focuses on the characteristics of multicultural adolescents in these family environments.

## 2. Materials and Methods

### 2.1. Research Design

This study included a secondary analysis of data from the Multicultural Adolescents Panel Study (MAPS) obtained by the National Youth Policy Institute [22] to verify how parental support mediates the bicultural acceptance attitude and self-esteem of multicultural adolescents.

### 2.2. Data Collection and Participants

The National Youth Policy Institute carried out a long-term panel survey focused on multicultural adolescents over a period of eight years, from 2011 to 2018. The data from 2018 utilized in this research are sourced from second-year high school students, aged 17, who were in fourth grade, at 11 years old, in 2011 when the National Youth Policy Institute study commenced. This research examined the latest data gathered from high school sophomores in 2018, marking the eighth instance of data collection.

### 2.3. Measurement of Variables

Bicultural Acceptance Attitude: The National Youth Policy Institute measured bicultural acceptance attitude using a modified evaluation scale [23]. Ten survey items were measured on a four-point Likert scale (1 = strongly disagree, 2 = disagree, 3 = agree, and 4 = strongly agree). Examples of questions include: “I enjoy Korean culture (music, movies, food, clothes, etc.)”, “I enjoy the culture of my foreign parent’s country (music, movies, food, clothes, etc.)”, “I am proud to live in Korea. I want to go to my foreign parent’s country and attend college or work there”. The higher the score, the more positive the attitude toward biculturalism. In this study, the Cronbach’s alpha was 0.78.

Parental Support: The National Youth Policy Institute measured parent support using a modified evaluation scale [24]. Nine survey items (for example, “My parents (or guardians) know and understand me well”) were measured on a five-point Likert scale (1 = strongly disagree, 2 = disagree, 3 = neither agree nor disagree, 4 = agree, and 5 = strongly agree). The higher the score, the greater the support children received from their parents. Examples of questions include, “Do my parents know and understand me well?”, “Do my parents give me advice that helps me study?”, and “Do my parents provide me with financial support?” In this study, the Cronbach’s alpha was 0.92.

Self-Esteem: The National Youth Policy Institute measured self-esteem using the Rosenberg Scale [6]. Nine survey questions were measured on a five-point Likert scale (1 = strongly disagree, 2 = disagree, 3 = neither disagree nor agree, 4 = agree, and 5 = strongly agree). Responses to negative questions were reverse-scored. Examples of questions include, “I think I have good character”, “I am generally satisfied with myself”, and “I have a positive attitude about myself”. A higher score indicates greater self-esteem. In this study, the Cronbach’s alpha was 0.88.

### 2.4. Data Analysis

The data in this study were analyzed using R statistical software (Version 4.2.0). The initial phase of data analysis included creating descriptive statistics for every variable. Each participant was characterized by their gender, age, the age of their mother and father, the education levels of their mother and father, and the countries of origin of both parents. The consistency of each measurement instrument was validated through Cronbach’s alpha. The data analysis is based on research by Baron and Kenny [25], employing hierarchical regression to determine if parental support acted as a mediator in the connection between bicultural acceptance attitudes and self-esteem among multicultural adolescents. At first, regression analysis was used to assess whether the independent variable (bicultural acceptance attitude) had a significant impact on the mediating variable (parental support). Following that, a multiple regression analysis was conducted to determine whether the independent variable (bicultural acceptance attitude) had a significant effect on the dependent variable (self-esteem). Lastly, when both the independent variable (bicultural acceptance attitude) and the mediating variable (parental support) were introduced, the analysis examined whether the mediating variable (parental support) influenced the dependent variable (self-esteem) and how the independent variable (self-esteem) changed in Step 1. Regression analysis was conducted to determine if the impact was reduced or eliminated. A bootstrap test (using the R statistical program) was performed to assess the statistical significance of the mediation effect. The method for calculating the mediating effect is as follows:

- Total effect (c):

(c): Y = cX + e1

- Relationship between independent variables and parameters (a):

M = aX + e2

- Relationship between parameters and dependent variables (b):

Y = c′X + bM + e3

- Indirect effect (a × b):

(a × b): a × b

- Direct effect (c′):

c′ = c − (a × b)

The number of bootstrap samples was set to 5000 and the results were deemed significant when no zeros were included between the lower and upper values. Bootstrapping is a statistical method for estimating data characteristics through sampling from a population. It is useful when trying to understand the distribution or uncertainty of data. The first step of this technique is to randomly extract samples from the original data, allowing for duplicates. The second step is to calculate the statistics of interest using the extracted samples. This process is repeated thousands of times to obtain the distribution of the statistics. Estimates of the distribution or parameters of the original data can be obtained by analyzing the distribution of the statistics. Bootstrapping provides reliable estimates and confidence intervals without making assumptions about the distribution of the data.

This study examined skewness and kurtosis, indicators commonly used to assess the normality of the data distribution. The analysis revealed that the observed variables in this study exhibited skewness ranging from a minimum of −0.037 to a maximum of 0.38 and kurtosis ranging from a minimum of 0.07 to a maximum of 2.58. These values met all the criteria, suggesting that the data followed a normal distribution.

### 2.5. Ethics Approval

Government approval for the use of the raw MAPS [22] data from the National Youth Policy Institute was obtained under the Regulations on the Disclosure and Use of Online Source Data for Multicultural Adolescents’ Health Development and Growth (no. 402003, 17 September 2021). Before initiating data analysis, this study was also approved by the bioethics committee of Dongyang University (no. 1041495-202111-HR-03-01). Prior to their participation, all participants provided informed consent to confirm their voluntary participation in the study. The principle of informed and voluntary consent was maintained, and all participants signed a consent form that explained their right to withdraw from the research at any time. Participant confidentiality was protected by omitting all personal identifying information.

## 3. Results

### 3.1. General Characteristics

Table 1 presents the general characteristics of the study participants. Young men represented 49.0% (*n* = 587) of the cohort, and young women represented 51% (*n* = 610). Most participants were aged 17 years (89.9%, *n* = 1064). The mean age of the adolescents’ mothers was 47.53 (SD = ±5.54) years and the adolescents’ fathers’ mean age was 53.22 (SD = ±4.59) years. Most parents of adolescents were high school graduates (mothers: *n* = 565, 47.2%; fathers: *n* = 594, 49.6%). Japan was the country of origin most often reported for mothers (*n* = 418, 34.9%) and Korea was the country of origin most often reported for fathers (*n* = 1107, 92.5%).

### 3.2. Descriptive Statistics and Correlation

Table 2 presents the degree of bicultural acceptance attitude, parental support, and self-esteem among the surveyed multicultural adolescents and the correlations between those variables. Bicultural acceptance attitude averaged 2.92 (±0.42) out of 4 points, parental support averaged 3.90 (±0.71) out of 9 points, and self-esteem averaged 3.71 (±0.34) out of 5 points. Positive correlations were observed between bicultural acceptance attitude and parental support (*r* = 0.438, *p* < 0.001), bicultural acceptance attitude and self-esteem (*r* = 0.144, *p* < 0.001), and parental support and self-esteem (*r* = 0.179, *p* < 0.001).

### 3.3. Mediating Effect of Parental Support on Bicultural Acceptance Attitude and Self-Esteem

**Figure 1:**
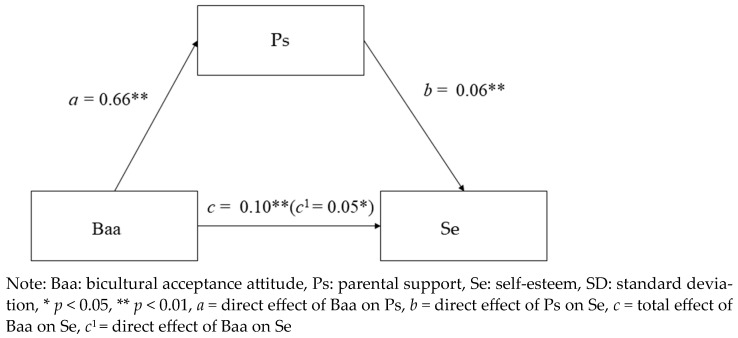
Mediating effect of parental support on the relationship between bicultural acceptance attitude and self-esteem.

Table 3 and Figure 1 presents the results showing the mediating effect of parental support on bicultural acceptance attitude and self-esteem. First, the independent variable bicultural acceptance attitude had a positive effect (*β* = 0.10, *p* < 0.001) on the dependent variable self-esteem. Second, the independent variable bicultural acceptance attitude had a positive effect (*β* = 0.66, *p* < 0.001) on the dependent variable parental support. Third, the independent variables bicultural acceptance attitude and parental support had positive effects on self-esteem (*β* = 0.05, *p* = 0.001). In other words, parental support was a mediating variable between the independent variable bicultural acceptance attitude and the dependent variable self-esteem.

Further analysis investigated the indirect effect (Table 3). The indirect effects of bicultural acceptance attitude and parental support on self-esteem were statistically significant (*β* = 0.05, *p* < 0.001). As a result of using the bootstrapping method to test the statistical significance of the mediating effect, the indirect effect of bicultural acceptance attitude and self-esteem did not include 0 between the lower and upper limits of the 95% confidence interval (0.021 to 0.07). As a result, parental support was found to have a partial mediating effect between bicultural acceptance attitude and self-esteem because both the direct (*β* = 0.10, *p* < 0.001) and indirect effects (*β* = 0.05, *p* < 0.001) of parental support on bicultural acceptance attitude and self-esteem were significant.

## 4. Discussion

Using Korean multicultural panel study data, this study investigated whether parental support was a mediating variable in the relationship between parental support and adolescent self-esteem in a sample of Korean multicultural adolescents. The study predicted that multicultural adolescents’ parental support would mediate the relationship between bicultural acceptance and parental support and that parental support would positively correlate with adolescent self-esteem. The results derived from this study are as follows:

First, parental support of multicultural adolescents positively affected their attitude toward accepting bicultural culture and their self-esteem. This result is in line with findings reported by Safa et al. [26] indicating a positive relationship between bicultural acceptance attitude and parental support. Research shows that although adolescents’ primary connection is shifting from parents to peers, parental support still plays a vital role in adolescent development [27]. In a study conducted by Lim, You, and Ha (2015) [28] parental support scores correlated positively with adolescent self-esteem in a Korean sample. Permissive parenting is considered the most suitable parenting style for adolescents’ self-esteem; adolescents’ self-esteem is higher in academic, emotional, familial, and physical aspects than authoritative parenting [29].

This finding supports other research results showing that social support—including parental support—has a positive effect on the life satisfaction, self-esteem, and achievement motivation of multicultural adolescents [30]. Previous studies have also demonstrated that the bicultural acceptance attitude of multicultural adolescents positively affects self-esteem [31,32]. The theory of cultural adaptation is a critical part of the linear concept of cultural adaptation, which views the culture of origin and mainstream culture as mutually exclusive. The former theory argues that both cultures can be positively accepted [33]. This is consistent with a previous study indicating that psychological adjustment increases with bicultural acceptance attitude [34].

Other research [35] reveals that bicultural acceptance increases self-esteem and psychological well-being. For example, in a sample of 123 Latino immigrant adolescents, they tended to associate biculturalism with positive self-perceptions of global self-worth. The self-esteem of adolescents with limited language skills was higher when they were with their families rather than with their friends [36]. Adolescents with more advanced language skills showed the opposite result. In terms of bicultural acceptance, it is evident that linguistic acculturation influences self-esteem.

Second, parental support mediates the relationship between bicultural acceptance attitude and self-esteem among multicultural adolescents. The bicultural acceptance attitude of these adolescents is ultimately the perception and attitude formed from interactions within their specific social environment [37]. Sullivan et al. [38] demonstrated that adolescents’ self-esteem is maintained when they exhibit integrated cultural adaptability rather than assimilation into the host culture. Adolescents who embrace integrated bicultural acceptance tend to have stronger bonds with their parents, which is a significant protective factor against adolescent issues. 

Their parents may influence the bicultural acceptance of adolescents. Hence, further exploration into the self-esteem of multicultural adolescents is warranted [35]. Biculturalism and familism are cultural assets associated with internalization problems and increased self-esteem. Studies provide valuable evidence indicating that family dynamics, including parent–child conflicts and familism, significantly influence the connection between acculturation and adolescent outcomes such as depression and self-esteem [39,40,41,42]. Parent–adolescent conflict was a decisive risk factor, which not only directly increased internalizing symptoms and self-esteem, but also mediated the effects of acculturation conflict and perceived discrimination on these outcomes. The findings indicated that familism is a cultural characteristic linked to a reduction in internalizing symptoms and an increase in self-esteem. Thus, the results revealed familism as a cultural trait associated with fewer internalizing symptoms and higher self-esteem. On the other hand, familism where family ties are strong showed the opposite result [35].

When multicultural adolescents adapt to the host culture, they do not change their cultural identity but rather develop a dual cultural identity that integrates multiple cultural values. Dual cultural identity can be linked to internalizing self-esteem [43,44]. Familism was identified as a cultural strength linked to a reduction in internalizing symptoms and an increase in self-esteem. In addition, adolescents’ involvement in American culture minimized internalizing symptoms; meanwhile, bicultural adolescents with higher engagement in their culture of origin reported higher self-esteem.

Other researchers have assessed predictors related to the self-esteem of Mexican immigrant adolescents and the moderating effects of family cohesion and parent–adolescent conflict on the relationship between the perception of social discrimination and Mexican immigrant adolescents’ self-esteem [42]. The study found that family cohesion was positively correlated with self-esteem, while parent–adolescent conflict and social discrimination were negatively correlated with self-esteem. Parental conflict moderated the relationship between perceived social discrimination and self-esteem. When adolescents experience conflict with their parents at home, it exacerbates the adverse effects of perceived social discrimination. Therefore, when teenagers face difficulties in various situations, it negatively impacts their self-esteem. This finding underscores the importance of identifying family-level mediators to assist young people in coping with negative social factors like discrimination [45]. Previous research with Chinese American adolescents found that family cohesion moderated the detrimental effects of discrimination on adolescents’ psychological well-being, whereas parent–adolescent conflict exacerbated these effects [46]. In a study involving 226 adolescent Latinos, 50% had a history of suicide attempts. Using path analysis, familism was revealed to be a cultural asset associated with lower levels of parent–adolescent conflict but higher levels of internalizing behavior. This study also found that self-esteem and internalizing behavior played a role in the relationship between parent–adolescent conflict and suicide attempts. This study highlights the importance of family involvement in culturally competent suicide prevention and intervention programs. Reducing parent–adolescent conflict and nurturing closer family relationships can also help boost self-esteem and decrease the likelihood of suicide attempts [47].

## 5. Conclusions

This study utilized MAPS data to examine the mediating role of parental support in the relationship between bicultural acceptance and self-esteem among multicultural adolescents. This discovery suggests that teenagers can enhance their ability to recognize their own values and understand themselves through an integrated sense of cultural acceptance, enhancing our comprehension of parental support in the relationship between bicultural acceptance and self-esteem among multicultural adolescents. The supportive role of parents is crucial in this process. To foster the healthy development of multicultural adolescents, parents need to engage with adolescents, and thus, policies and strategies to bolster the parent–child relationship should be encouraged. Further research is necessary to enhance our knowledge to effectively assist parents in enhancing adolescents’ self-esteem. To enhance the mental well-being of multicultural adolescents, educational institutions and local governments should implement school support programs in schools and establish parental mentoring groups. Additionally, they should provide multicultural training for teachers who work with adolescents and reinforce academic support within schools and communities to enhance interactions with adolescents. Initiatives should also be undertaken to promote supportive parenting behaviors.

## 6. Limitations

The limitations of this study are as follows: The study’s cross-sectional design hinders the ability to draw conclusions about causal relationships. The sample predominantly consists of Asian multicultural parents and their children, indicating potential variations from the traits of multicultural families in different regions.

However, given the limitations of the MAPS data, the specific characteristics of the multicultural adolescents included in the sample could not be determined. Moreover, clearly establishing variables was complicated. The resulting interpretation might need to be revised in the context of a lack of concrete knowledge about the growth of adolescents with a bicultural background in South Korea. Parental unity, number of siblings, chronic diseases of adolescents, and neurodevelopmental problems enhance the study’s robustness. However, limitations exist as they cannot be included due to restricted variables. Future research should include a detailed study into how the bicultural acceptance attitude of multicultural adolescents affects the development of those multicultural adolescents within the structure and dynamics of multicultural families.

## Figures and Tables

**Table 1:** General characteristics of a cohort of multicultural adolescents.

Category		*N*	%	Mean ± SD
Sex	Male	587	49.00	
	Female	610	51.00	
Age	16 Years	90	7.50	
	17 Years	1064	88.90	
	18 Years	39	3.20	
	19 Years	3	0.30	
	20 Years	1	0.10	
Mother’s age				47.53 ± 5.54
Father’s age				53.22 ± 4.59
Mother’s education	≤Middle school	133	9.40	
	High school	565	47.20	
	University (two years)	303	25.30	
	University (four years)	189	15.80	
	Graduate school	5	0.40	
	Unknown	22	1.90	
Father’s education	≤Middle school	360	30.10	
	High school	594	49.60	
	University (two years)	70	5.90	
	University (four years)	113	9.50	
	Graduate school	10	0.80	
	Unknown	50	4.10	
Mother’s country of origin	Korea	39	3.20	
	China (Han Chinese)	84	7.10	
	China (Korean Chinese)	211	17.60	
	Vietnam	25	2.00	
	The Philippines	311	26.10	
	Japan	418	34.90	
	Thailand	49	4.10	
	Others	60	5.00	
Father’s country of origin	Korea	1107	92.50	
	China (Han Chinese)	2	0.20	
	China (Korean Chinese)	1	0.08	
	Vietnam	2	0.10	
	The Philippines	4	0.30	
	Japan	16	1.30	
	Thailand	1	0.08	
	Others	14	1.20	

Note: SD; standard deviation.

**Table 2:** Degrees of bicultural acceptance attitude, self-esteem, and parental support with their corresponding correlations.

Variable	Mean ± SD	Skewness	Kurtosis	*r* (*p*)		
				Baa	Ps	Se
Baa	2.92 ± 0.42	0.07	0.82	1	—	—
Ps	3.90 ± 0.71	−0.037	0.07	0.438 **	1	—
Se	3.17 ± 0.34	0.3 8	2.58	0.144 **	0.179 **	1

Note: Baa; bicultural acceptance attitude, Ps; parental support, Se; self-esteem SD; standard deviation; ** *p* < 0.01; *r* = correlation effect.

**Table 3:** Mediating effect of parental support on the relationship between bicultural acceptance attitude and self-esteem.

Independent Variable	Dependent Variable	Β	SE	t(*p*)	Adj. R^2^	F(*p*)	Indirect Effect
							*β*(*p*)
Baa	Se	0.10	0.02	5.038 **	0.01998	25.38 **	-
Baa	Ps	0.66	0.039	16.88 **	0.1919	284.9 **	-
Ps	Se	0.06	0.01	4.554 **	0.0359	23.27 **	0.05 **
Baa		0.05	0.02	2.56 *			

Note: Baa: bicultural acceptance attitude, Ps: parental support, Se: self-esteem, SD: standard deviation; * *p* < 0.05; ** *p* < 0.01; *β*: coefficient, SE: standard error.

## Data Availability

The data of this manuscript is available from the corresponding author upon a reasonable request.
